# Risk factors for MOH and chronic daily headache: an 11-year follow-up study. The Nord-Trøndelag Health Studies (HUNT)

**DOI:** 10.1186/1129-2377-14-S1-P13

**Published:** 2013-02-21

**Authors:** K Hagen, M Linde, TJ Steiner, JA Zwart, LJ Stovner

**Affiliations:** 1 Department of Neuroscience, Norwegian University of Science and Technology, Norway; 2 Department of Neuroscience, Norwegian University of Science and Technology, UK; 3 Oslo university Hospital, Norway

## Background

Medication-overuse headache (MOH) is relatively common, but its incidence has not been calculated and there are no prospective population-based studies that have evaluated risk factors for developing MOH. Aim of the study: This was to estimate incidences of and identify risk factors for developing chronic daily headache (CDH) and MOH.

## Method

This was a longitudinal population-based cohort study using data from the Nord-Trøndelag Health Surveys performed in 1995-1997 and 2006-2008.

## Results

Among the 51,383 participants at baseline, 41,766 were eligible approximately 11 years later. There were 26,197 participants (responder rate 63%), among whom 25,596 did not report CDH at baseline in 1995-1997. Of these, 201 (0.8%) had MOH and 246 (1.0%) had CDH without medication overuse (CDHwoO) 11 years later. The incidence of MOH was 0.72 per 1000 person-years (95% confidence interval 0.62-0.81). In the multivariate analyses, a 5-fold risk for developing MOH was found among individuals who at baseline reported regular use of tranquilizers [odds ratio 5.2 (3.0-9.0)] or who had a combination of chronic musculoskeletal complaints, gastrointestinal complaints, and Hospital Anxiety and Depression Scale score ≥11 [odds ratio 4.7 (2.4-9.0)]. Smoking and physical inactivity more than doubled the risk of MOH. In contrast, these factors did not increase the risk of CDHwoO.

## Conclusion

In this large population-based 11-year follow-up study, several risk factors for MOH did not increase the risk for CDHwoO, suggesting these are pathogenetically distinct. If the noted associations are causal, more focus on comorbid condition, physical activity, and use of tobacco and tranquilizers may limit the development of MOH.
